# Ultrasound-Guided (USG) Aspiration From Sacroiliac Joint in Tuberculosis Is Rare: An Atypical Case Report From a Developing Country

**DOI:** 10.7759/cureus.83834

**Published:** 2025-05-10

**Authors:** M Masudul Hassan, Mohammad Abul Kalam Azad, Md Nahiduzzamane Shazzad, Syed Jamil Abdal, Farhana Binty Rashid

**Affiliations:** 1 Rheumatology, Bangabandhu Sheikh Mujib Medical University (BSMMU), Dhaka, BGD; 2 Obstetrics and Genecology, Dhaka Medical College, Dhaka, BGD

**Keywords:** genexpert, genexpert assay, mycobacterium, rifampicin sensitive, sacroiliitis, tuberculosis, tuberculous sacroiliitis, ultrasonographic guided aspiration, usg aspiration

## Abstract

Tuberculosis (TB) in the musculoskeletal system is rare, though it is an emerging health concern in many developing countries. Sacroiliac joint (SIJ) TB is uncommon. This report highlights the use of ultrasound-guided (USG) aspiration to diagnose SIJ TB. We report a 23-year-old boy who presented with low back pain and localized tenderness over the SIJ. Initial imaging showed signs of inflammation. USG aspiration was performed successfully to obtain fluid for microbiological analysis, and laboratory investigations confirmed the presence of *Mycobacterium tuberculosis*. Sacroiliac TB often presents in the absence of classic symptoms, and minimally invasive procedures like USG aspiration can play a role in providing rapid diagnosis and treatment. Early identification and appropriate management can prevent further complications and improve patient outcomes in regions where TB remains endemic in the world.

## Introduction

Tuberculous sacroiliitis is rare. Musculoskeletal (MSK) tuberculosis (TB) accounts for 5%-10% of all cases of TB [1]. The symptoms are not specific [2], and spondylarthritis is among the differential diagnoses [3]. The diagnosis of sacroiliac joint (SIJ) pathology is done by MRI [3], but CT or ultrasound-guided (USG) aspiration from the SIJ is essential for diagnosis [4]. In CT-guided aspiration is costly and adynamic, but USG is less costly with dynamic maneuver and is essential to confirm TB. AFB staining with GeneXpert (Cepheid, Sunnyvale, CA) from aspirated material confirms the diagnosis. We report a case of a 23-year-old Bangladeshi student presenting with pain in the lower back area. USG reveals a hypoechoic area in the left sacroiliac area. Aspiration from the hypoechoic area was done. *Mycobacterium tuberculosis* was diagnosed by AFB stain with GeneXpert. The patient improved with nine-month antitubercular treatment with surgical drainage of the abscess. USG aspiration followed by AFB stain and GeneXpert has shown an accurate diagnosis of SIJ TB. The advantage of USG needle aspiration is that it is cost-effective and widely available in developing countries like Bangladesh. Despite its utility, USG aspiration is limited in clinical literature, especially from developing countries.

## Case presentation

A 23-year-old Bangladeshi man presented with severe low back pain for 1.5 months. The pain was insidious in onset, gradually progressive, with morning stiffness. He also complained of difficulty walking due to back pain. He had a considerable weight loss of 10 kg within 1.5 months. He had low-grade fever with night sweats and fatigue. He had no history of contact with the tubercular patient or family history of TB. There is no history of trauma, but he traveled from Bangladesh to Australia prior to the diagnosis of TB. The patient had no history of pain in any other joint and no history of loose motion, diarrhea, or painful red eye.

Examinations showed the temperature was 99-degree Fahrenheit, and other vital signs were normal. Fundoscopy was normal. Active and passive range of movement was difficult due to pain and tenderness in the left SIJ. The Schober test was < 5 cm, which was positive, and the flexion abduction and external rotation (FABER) test was positive on the left side of the SIJ.

Straight leg rising (SLR) 60 °, heel walking, and toe walking were normal. Examination of other joints and systemic examination were normal. Our differential diagnosis was axial spondylarthritis (SpA) and tuberculous infection in the SIJ. Points in favor of axial SpA are inflammatory LBP and the absence of other features of axial SpA. Again, points in favor of infection in the SIJ include pain in the left SIJ and significant weight loss with low-grade fever. The investigation showed that Hb, the total count of RBC, and WBC, with differential count, were normal. Initial laboratory findings of the patient are shown in Table 1.

**Table 1:** Initial laboratory findings of the patient RBC – Red Blood Cell, WBC – White Blood Cell, ESR – Erythrocyte Sedimentation Rate, SGPT – Serum Glutamic Pyruvic Transaminase, HBsAg – Hepatitis B Surface Antigen, RA – Rheumatoid, HLA – Human Leucocyte Antigen, Anti-CCP – Anti-cyclic Citrullinated Peptide, MT – Mantoux Test, TSH – Thyroid-Stimulating Hormone

Name of the test	Laboratory findings	Normal range
Hemoglobin (g/ dL)	12.2	12-15
Total count of WBC (c mm of blood)	6,990	4,000-1,100
Total count of RBC (c mm of blood)	4.39×10^9^	4.5×10^9 ^- 6.5×10^9^
Total platelet count (c mm of blood)	3.00 ×10^9^	1.5- 4.5 ×10^9^
Neutrophil (%)	65	40-75
Lymphocyte (%)	24.3	20-40
Eosinophil (%)	4.9	2-6
Monocyte (%)	4.9	2-10
ESR (mm in the first hour)	80	0-10
C-reactive protein (mg/L)	31	<6
Serum creatinine (mg/dL)	0.82	0.7-1.3
SGPT (U/mL)	130	<40
Alkaline phosphatase (U/L)	110	30-130
HBSAg	Negative	Normal
Anti-Hbc total	Negative	Normal
Anti-HCV	Negative	Normal
RA (IU/mL)	Negative	<15.7
Anti-CCP (U/mL)	0.5	< 5
HLA B 27	Negative	Normal
Anti-HIV-1 and 2	Negative	Normal
MT (mm after 72 hours)	3	>10
QuantiFERON Tb Gold plus	Negative	Normal
Vitamin D (ng/mL)	12.1	30-100
TSH (mIU /mL)	3	0.35-5.5

**Figure 1:**
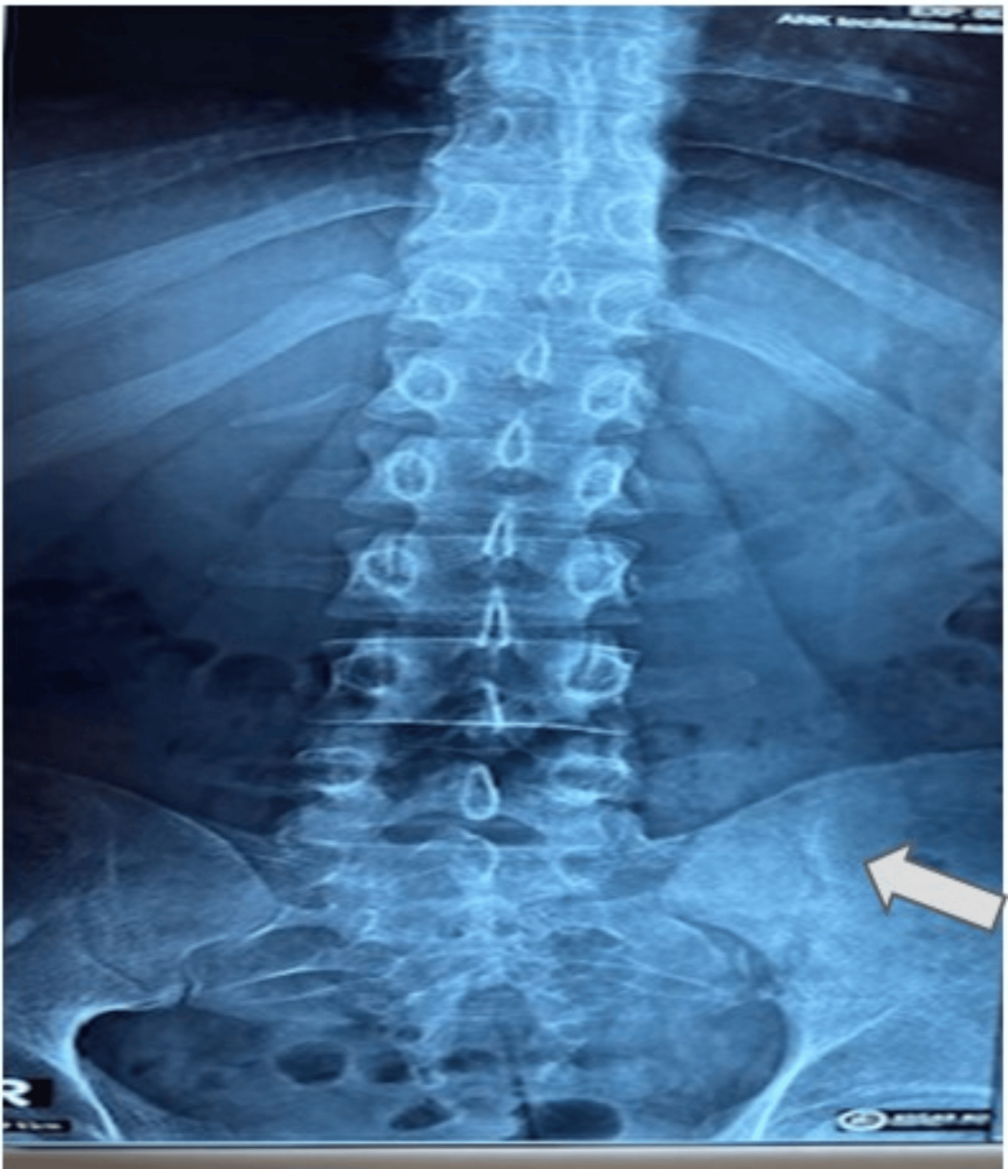
X-ray of lumbosacral spine showing hazy left-sided sacroiliac joint, sclerosed with irregular margin suggestive of left-sided sacroiliitis

**Figure 2:**
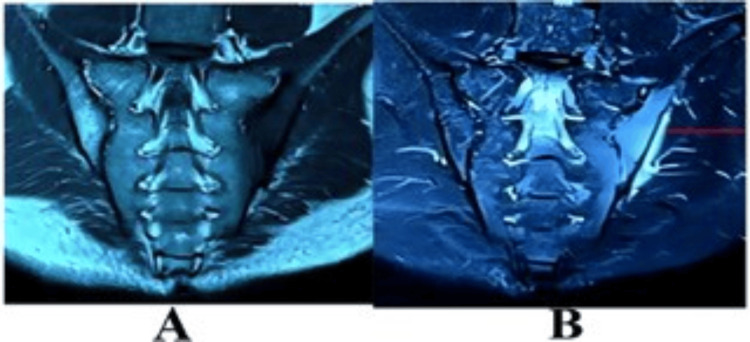
MRI of sacroiliac joint. (A) T1 phase, (B) T2 phase left-sided sacroiliitis. MRI of sacroiliac joint showing hypo-intense signal in the sacroiliac joint, more marked on the iliac side, and also extending to the dorsal surface of the sacrum in T1 phase and hyperintense in the T2 phase.

MRI of the SIJ showed evidence of an ill-defined area of altered signal intensity having predominant T2 hyperintensity in the left SIJ, features suggestive of left-sided sacroiliitis with periarticular abscess (Figures 2A, 2B).

**Figure 3:**
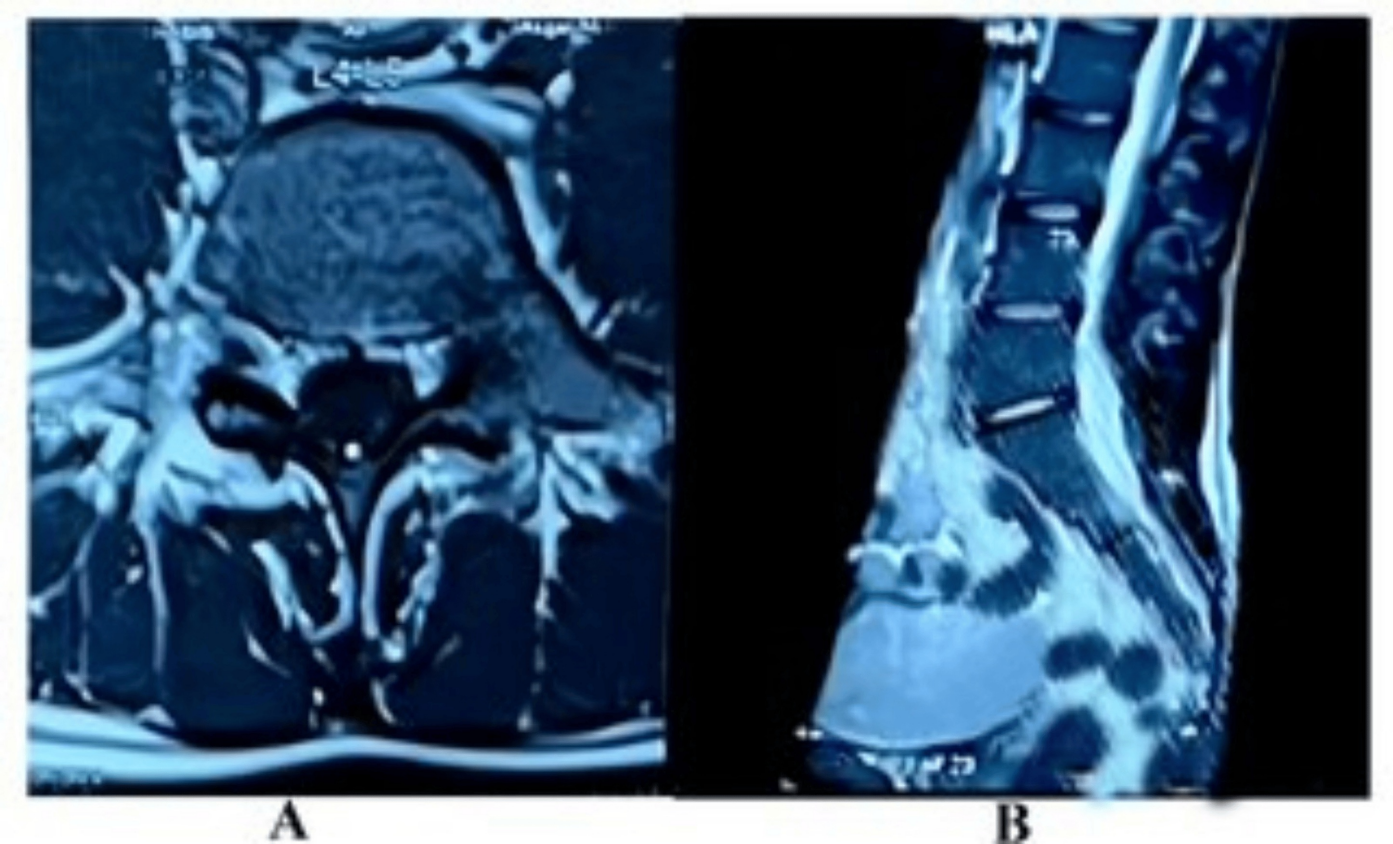
MRI of lumbosacral spine. (A) Transverse view T2 phase, (B) longitudinal view T2 phase. MRI of the lumbosacral spine showing normal findings in transverse and longitudinal views.

MRI of the lumbosacral spine was normal (Figures 3A, 3B).

**Figure 4:**
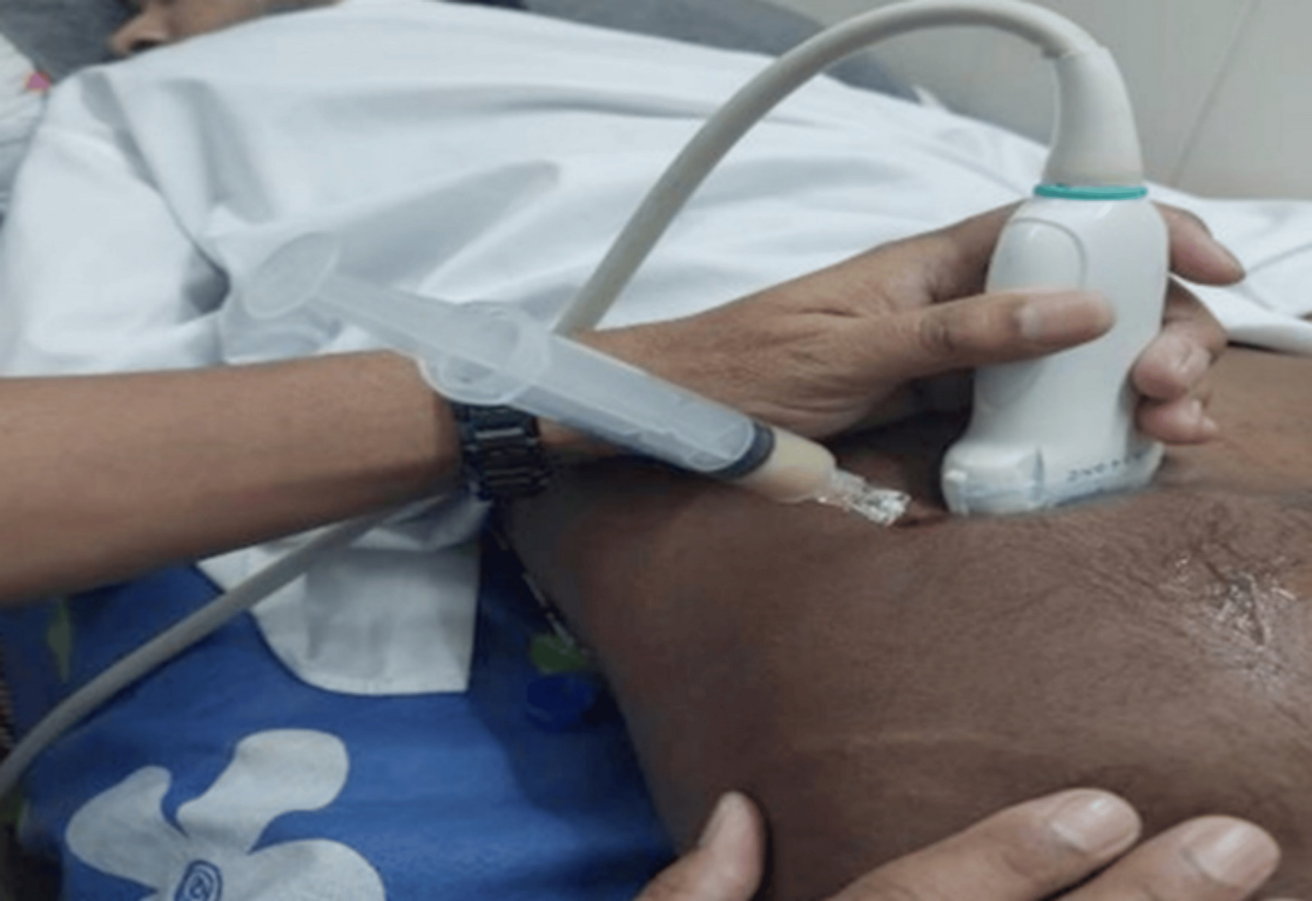
USG aspiration from the sacroiliac area.

USG of SIJ with Mindray, 14-6 Hz probe showed a large, about 2 to 2.5 cm mixed density hypoechoic area in the left SIJ. Ultrasonographic-guided aspiration was done with all aseptic precautions, and creamy pus came out (Figure 4).

On examination of pus, AFB staining was positive, and GeneXpert ultra for *M. tuberculosis* was also positive with sensitivity to rifampicin. He was treated with a four-drug anti-tubercular therapy. Treatment regimen of two months of four drug with isoniazid (4-6 mg/kg once daily), rifampicin (8-12 mg/kg /day), pyrazinamide (20-30 mg/kg/day), and ethambutol (15-25 mg/kg/day) followed by seven months of isoniazid and rifampicin. His ESR and CRP were normal after starting antitubercular therapy, and the x-ray finding of SIJ also improved and marked improvement in well-being and significant weight gain within six months.

## Discussion

TB in the SIJ is rare. The incidence of MSK TB is 5%-10% in general worldwide [1,2]. The incidence of TB is rising in developing countries in Asia and also in Africa [3]. We reported an atypical case of left-sided tuberculous sacroiliitis, presenting with severe low back pain with constitutional symptoms. It is reported by Vatra [5] that a definite diagnosis of tubercular sacroiliitis is essential to identify acid-fast bacilli (AFB) from the lesion of the SIJ. A family history of contact TB may be helpful in the diagnosis, but our patient had no history of TB in his family. Our patient’s tuberculin test was negative with an induration of only 03 mm, and it is not diagnostic. It is also reported by Dodd that the specificity of the tuberculin test is low in a high-prevalence country of TB [6].

Very high ESR and CRP are the nonspecific inflammatory markers and therefore cannot be diagnostic of anything. They merely reflect a high inflammatory state. It is also helpful in assessing the response to treatment [7]. The ESR of our patient decreased significantly after the initiation of anti-tuberculous therapy.

In the early stage of sacroiliac TB, the x-ray of the joint is normal. An ill-defined joint line is an early feature of tuberculous sacroiliitis [8]. It has been shown that the importance that CT scan studies, which delineate the extent of joint destruction, and MRI can delineate the abscess in the soft tissue [9]. In our case, the x-ray of the pelvis demonstrated haziness with an ill-defined margin in the left SIJ, indicating sacroiliitis. MRI shows an abscess in the soft tissues of the left SIJ area. 

The prevalence of pulmonary TB with tubercular arthritis is approximately 50% [10]. In our case, we found no TB feature in other sites. We did USG-guided aspiration and found mycobacteria by Ziehl-Neelsen staining and positive GeneXpert with sensitivity to rifampicin. A positive GeneXpert test, combined with a positive Ziehl-Neelsen stain, has been reported to provide microbiological confirmation of TB [10].

It is said that positive GeneXpert of TB and Ziehl-Neelsen staining should be considered microbiological confirmation of TB. In the study by Held et al., the sensitivity of GeneXpert in detecting TB in both HIV-positive and HIV-negative patients was reported to be 96.9% (31/32) and 89.6% (43/48), respectively [11]. It is challenging to diagnose articular TB without another focus of TB [5]. Identification of *M. tuberculosis* was essential, because it was suspicious both in SpA and TB in the left SIJ. Osteochondroma is the differential diagnosis of unilateral sacroiliitis. So, CT-guided biopsy is needed for a definite diagnosis. It was reported that atypical features of TB mimicking a tumor are rare in a few cases [12]. Appropriate biopsy and culture are essential to rule out neoplastic diseases, but in our case, we deferred because of a positive GeneXpert. There is often a delay in the diagnosis of tuberculous arthritis, because of features TB are less or sometimes ignore the feature of TB.

It is reported in the study that bilateral and symmetrical distribution of sacroiliitis is usually seen in ankylosing spondylitis, whereas unilateral abnormalities are the most typical feature in infection [13]. Our case revealed unilateral left sacroiliitis and was also diagnosed relatively early, within two months. Still, the mean duration of diagnosis was reported to be 16 to 19 months [7]. Treatment of pulmonary and extra-pulmonary TB same drug, but the period of extra-pulmonary TB may extend up to 36 months [14]. Most of the patients respond well to drug therapy, and surgery is rarely required [5], but our patients responded well to anti-tubercular treatment with surgical drainage.

## Conclusions

USG aspiration from the SIJ in TB cases is a rare and valuable diagnostic tool. This report highlights the successful application of USG-guided aspiration from SIJ. Using US guidance allows for accurate aspiration, minimizing complications and improving outcomes. Early diagnosis and intervention are essential for managing SIJ TB, and such minimally invasive procedures can play an important role in improving patient care. Conservative treatment with antitubercular agents ensures a full recovery in early disease. Abscesses should be drained surgically if they are deep-seated and large.
